# Insights from the comparative genome analysis of natural rubber degrading *Nocardia* species

**DOI:** 10.6026/97320630017880

**Published:** 2021-10-31

**Authors:** Biraj Sarkar, Aayatti Mallick Gupta, Sukhendu Mandal

**Affiliations:** 1 Laboratory of Molecular Bacteriology, Department of Microbiology, University of Calcutta, 35, Ballygunge Circular Road, Kolkata, 700019, India; 2 Department of Chemical, Biological & Macro-Molecular Sciences, S. N. Bose National Centre for Basic Sciences, Block-JD, Sector-III, Salt Lake, Kolkata-700 106, India

**Keywords:** poly-cis-isoprene, rubber degradation, *Nocardia*, whole genome analysis, phylogenomics

## Abstract

*Nocardia* are known to be a facultative human pathogen and can cause infection in immune compromised patients. Though the details research on the virulence factors of *Nocardia* are scanty but numerous genes that code such factors were reported from different species of *Nocardia*. Despite of the presence of several virulence factors, species of this genus have been shown to have role in remediation of many toxic and hazardous materials from the environment. In this study, genome sequences of rubber degrading *Nocardia* sp. BSTN01 and N.nova SH22a have been analyzed to locate the potential virulence genes. Also, the genomes of facultative pathogenic *Nocardia* like, *N.africana*, *N. brasiliensis*, *N. kruczakiae*, *N. transvalensis* and *N. veterana* have been analyzed to find the gene encoding latex clearing protein (Lcp), a rubber oxygenase enzyme of Gram-positive action bacteria. The study provides an insight about the potentiality of rubberdegrading *Nocardia* species to emerge as future human pathogens and also the probability of a serious concern if the studied facultative pathogens of *Nocardia* like *N. africana*, *N. brasiliensis*, *N. kruczakiae*, *N. transvalensis* and *N. veterana* are capable of degrading rubber, a regularly used material in clinics. Moreover, use of such possible pathogenic strains for their known role in bioremediation of rubber waste from the environment might be deleterious.

## Background:

The genus *Nocardia* consists of aerobic, filamentous, Gram-positive,non-motile actinomycetes which have been reported to be isolated from a diverse range of environments around the globe [1-3]. Various *Nocardia* spp. are now known to be facultative intracellular human pathogens which were reported to be capable of causing localized or disseminated infection in both immune competent and immune compromised humans and in animals [3-5]. Although infection causing *Nocardia* are generally known to be opportunistic and infection occurs in immune-compromised patients, around 15% of such infections do not even exhibit any definable predisposing condition [6,7]. According to a report published in 2017, out of 92 recognized species of *Nocardia*, listed in LPSN (the List of Prokaryotic names with Standing in the literature), 54 species have been found to be clinically relevant [8]. Moreover, the number of reported cases of *Nocardia* infections have been increasing since last few decades [9,10], which is indeed a matter of great concern in clinical relevance. Numerous cases have been reported where infections caused by *Nocardia* were found to affect lungs, central nervous system, skin and othervital organs. Nonetheless, *Nocardia* can also possess serious life-threatening disseminated infections like osteomyelitis and *Nocardia*l sepsis [10-12]. Studies involving genomics helps in understanding the diversity, taxonomy and evolutionary relatedness of species belonging to the genus *Nocardia* [13]. Multiple genome sequences of different species of *Nocardia* have been reported till date. These sequences provide insights about different virulence factors which are known to play crucial role in cell invasion, modulation of phagocyte functions and survival inside the host macrophages resulting successful pathogenesis [14-19]. Not just the genomes of pathogenic species of *Nocardia*, but species of *Nocardia* that are known to be endophytes, has also been studied to explore their biological roles [20,21]. Reports involving studies on the whole genome sequence (WGS) also provided insights into the molecular basis of resistance towards different antibiotics exhibited by different species of *Nocardia* [22-25]. Studies on the WGSs of N. cyriacigeorgica strains helped in understanding the physiology, evolution and adaptation of the environmental bacteria for being converted into a pathogenic one [26,27]. Genome based analysis of different species of *Nocardia* revealed some of them to harbour genes responsible for the production of important secondary metabolites like polyketide synthase (PKS-I) and non-ribosomal peptide synthetases (NRPS) [28,29]. Species belonging to the genus *Nocardia* were also reported to produce valuable metabolites like antibacterial compounds, UV-protectant molecules, immune suppressant, nocavionin and nocobactin derivatives with anti muscarinic activities [30-33]. On the other hand, some species belonging to the genus *Nocardia* have been reported to be capable of degrading varied kinds of environmental toxic and hazardous hydrocarbon-based materials, including rubber [34-37]. Such behaviour exhibited by these Gram-positive actinomycetes is attributed to their ability to use the polyisoprene backbone of rubber as a source of carbon for their growth and proliferation in scarcity of other easily accessible nutrients. Several studies reported that these group of bacteria are capable of extracellular cleaving the high-molecular-weight polyisoprene backbone of rubber by virtue of their extracellularly secreted latex clearing protein, Lcp (EC 1.13.11.87), which in turn allows the bacteria to easily uptake the resultant low-molecular-weight oligoisoprenoids into the cell for further metabolic processes required in the quest of energy and survival [36,38-40]. All rubber degrading actino bacteria are known to harbour one or more lcp gene in their genome or plasmid which encodes the latex clearing protein [38]. The functionality of lcp gene and its enzyme product were analysed using by means of gene deletion, cloning and protein expression studies [40-43].

Numerous polymer materials which are chiefly made of rubber are extensively used in the field of clinical science, prosthetics and hygiene products. As examples, urinary catheters and gloves used clinically are generally made up of rubber [44-46], gutta-percha which is basically a trans- 1,4-polyisoprene form of rubber has been widely used for years in endodontics [47] and products like menstrual cups are also primarily composed of rubber which comes under the category of feminine hygiene products [48]. Interestingly, *Nocardia* species can colonize and produce biofilm on clinical or hospital resources that are primarily composed of rubber or poly-isoprene. Such materials can put the treated patients under serious conditions causing *Nocardia*-bacteremia. Such an instance is stated in case of*N. nova* complex, as it was capable of forming biofilm in polyisoprene made catheters, like central venous catheters which are normally needed by cancer patients [49]. According to a study performed at the University of Texas M.D. Anderson Cancer Center, around 7% of all invasive infections caused by *Nocardia* had central venous catheters in common [50]. Therefore, the comparative genome analysis of an unreported rubber degrading *Nocardia* (i) *Nocardia* sp BSTN01 and (ii) a reported efficient rubber degrading *N. nova* SH22a have been analysed in to find potential virulence factor in the genome. We have also analysed the complete genome sequences of other related bacteria for lcp gene in their genome. This data will help us to know the aspect of emerging infections caused by rubber degrading species of *Nocardia*, particularly for environmental remediation without knowing their pathogenicity attributes.

## Materials & Methods:

### Retrieve of genomic data of *Nocardia* spp:

In this study the WGS sequence of BSTN01 was compared with the complete and WGS sequences of 6 other different species of *Nocardia*which have been obtained from NCBI database. A list of organisms with their accession number has been tabulated in Table 1(see PDF).

### Phylogenetic analysis:

Phylogenetic analysis of the WGS and complete genomesequences used in this study is inferred with REALPHY 1.12 [51]. It employs Maximum likelihood algorithm with the general time reversible (GTR) of the nucleotide substitution model with gamma distribution (G) to construct the tree. The resultant tree is visualized and annotated in FigTree (http://tree.bio.ed.ac.uk/software/figtree/).The average nucleotide identity (ANI) was calculated in EzGenome (https://www.ezbiocloud.net/taxonomy) [52].

### Annotation of the genomes

In order to locate the lcp gene and different virulent factors (VFs) from the WGS of the selected *Nocardia* spp., the genome sequences of respective strains were annotated in the RAST (Rapid Annotations Using Subsystems Technology) server [53] and PATRIC bioinformatics resource centre (https://patricbrc.org/app/Annotation), an online platform [54].

### Comparison of Lcp homologues:

LCP homologues are identified and multiple sequence alignment (MSA) using clustal omega (https://www.ebi.ac.uk/Tools/msa/clustalo/) of all the sequences were carried out.

### Pathogenomic analysis

To identify the virulence factor within various species of *Nocardia*, standard virulence factors of Mycobacterium tuberculosis has been used as reference. Genes homologous to those of *M. tuberculosis* with functions related to stress adaptation, phagosome arresting, nitrate reductases, secreted protein, effector delivery system and mammalian cell entry (mce) have been harnessed using VFDB database [55]. *M. tuberculosis* is a well-studied known species of pathogenic actinobacteria. As both *Nocardia* and *M. tuberculosis* are from the same phylum, in this study the genes responsible for encoding the virulence factors in *M. tuberculosis* were searched in the genomes of selected species of *Nocardia*. All the above-mentioned virulence factors from the genome sequences of *Nocardia* have been retrieved further fromRAST (Rapid Annotations Using Subsystems Technology) server [53] and PATRIC bioinformatics resource centre (https://patricbrc.org/app/Annotation) [54].BLAST of the amino acid sequences of the VF genes from the different *Nocardia* species used in this study were also performed against the *M. tuberculosis* (NCBI taxonomy ID: 1773) using Basic Local Alignment Tool (BLASTp).

## Results and Discussion:

*Nocardia* sp. strain BSTN01 is an isolate capable of degrading both synthetic and natural rubber, and was isolated from the water stored in latex collecting cup from the wastes of a rubber processing unit. The whole genome shotgun sequence of the strain is submitted in NCBI under the accession number NZ_JADKYP010000000. Another well-established rubber degrader *N. nova* SH22a is known to be capable of degrading both cis-isoprene rubber (NR) and trans-isoprene rubber (gutta-percha) [36]. Studies has already confirmed the presence of lcp gene encoding the latex clearing protein (Lcp) which is the primary and most vital enzyme involved in the oxidative cleavage of the polyisoprene backbone in the process of biodegradation in both BSTN01 and SH22a [36]. These strains were isolated from different geographical locations and yet had not been observed or reported for exhibiting virulence in humans. There are numerous pathogenic and facultative pathogenic *Nocardia*known to cause health issues in humans. For instance, *N. veterana*f NBRC 100344 is an actinomycete which was isolated from the bronchoscopic lavage of a 78-year-old patient with a past history of tuberculosis pleurisy exhibited bilateral upper lobe lesions [56]. *N. africana* NCTC13184 is such a pathogen which was isolated from patients with pulmonary infections [57]. *N. kruczakiae* was found to be responsible for causing traumatic endo phthalmitis in a patient with eye injury. Strains of *N. kruczakiae* are known to be opportunistic pathogens infecting immune compromised patients [58,59]. Similarly, there are reports of mycetoma caused by N. transvalensis and *N. brasiliensis* [60,61].

## Segregation of rubber degrading *Nocardia*

From the phylogenetic analysis of the selected species of *Nocardia*, it was found that *N. kruczakiae* NBRC 101016 and strain BSTN01 share a common clade (Figure 1 - see PDF), indicating the strains to be descended from a common ancestor. But as the average nucleotide identity between the two strain is 94.56% (Table 1 - see PDF), which is well below the cut-off level (95-96%) recommended as the ANI criterion for interspecies identity [62], the strain BSTN01 does not belong to the same species as strain NBRC 101016. In fact, the strain BSTN01 should be a distinct species with respect to the rest of the considered species of *Nocardia* in this study according to the ANI criterion for interspecies identity [62]. Moreover, even *N. nova* SH22a is placed in a distinct clade in the phylogenetic tree which indicates both the strains BSTN01 and SH22a to be distantly related.

## The Rubber degrading genes of *Nocardia*are conserved:

Rubber degrading actinobacteria produces latex clearing protein (Lcp) encoded by the lcpgene. This latex clearing protein is in fact a rubber oxygenase which initiates the degradation of rubber by cleaving the polyisoprene backbone extracellularly, which in turn allows the easy uptake of low molecular weight oligo-isoprenoids [38-40]. The amino acid sequences of latex clearing proteins (Lcp) from different species are known to be related and all are found to share a common domain of unknown functioni.e., DUF2236 together with other hypothetical proteins with diverse functional annotations [40,63,64]. From the translated CDs' of WGS sequences of the *Nocardia* spp. considered in this study, it was found that, *Nocardia* sp. BSTN01 harbours 10 DUF2236 sequences; whereas, *N. nova* SH22a, *N. africana* NCTC13184, *N. brasiliensis* ATCC 700358, *N. kruczakiae* NBRC 101016, N. transvalensis NBRC 15921 and *N. veterana* NBRC 100344 harbours 12, 8, 11, 13, 6, and 5 DUF2236 sequences, respectively. The DUF2236 amino acid sequences of BSTN01 showing highest similarity with the already proven LcpSH22a (WP025350295) from *N. nova* SH22a were considered for MSA analysis (Figure 2 - see PDF). According to the percentage identity matrix performed for the considered DUF2236 sequences employing the online platform of https://www.ebi.ac.uk/Tools/msa/clustalo/, WP194807853 (from *Nocardia* sp. BSTN01) showed 85.43% identity with respect to the WP025350295 (from *N. nova* SH22a); whereas, the WP040717816 (from *N. veterana* NBRC 100344), WP062963305 (from *N. africana* NCTC13184), WP063012276 (from *N. kruczakiae* NBRC 101016), WP041562627 (from *N. brasiliensis* ATCC 700358) and WP040746909 (from N. transvalensis NBRC 15921) exhibited 87.41%, 83.46%, 85.19%,66%, and67% of identity, respectively with WP025350295. This high percentage of identity amongst the candidate sequences and the Lcp protein of *N. nova* SH22a indicates that, all the considered strains might be capable of degrading polyisoprene, provided the environment they are growing on are otherwise favourable.

From the physical maps (Figure 3 - see PDF) of the studied species of *Nocardia*, it was found that the rubber degrading *Nocardia* sp. BSTN01 and three others studied *Nocardia* strains, i.e., NBRC 15921, NCTC1314 and NBRC 101016 harbours similar genes downstream to their Lcp encoding gene. The downstream of lcp were followed by genes encoding a FAD:protein FMN transferase, a putative Fe-S, FMN containing oxidoreductase, further followed by two yrbE genes (yrbEA and yrbEB) and mammalian cell entry (mce) cluster (Figure 3a, c, d and e - see PDF). Interestingly, it has already been proposed that the transport of low-molecular-weight oligoisoprenoids formed due to the extracellular oxidative cleavage of polyisoprene by latex clearing protein (Lcp) into the cytoplasm via an ATP-dependent, Mce protein-driven mechanism [38]. Generally, the mce clusters have similar structures comprising of two yrbE genes (yrbEA and yrbEB), encoding integral membrane protein, followed by mammalian cell entry (mce) genes mceA, mceB, mceC, mceD, mceEandmceF. The mce clusters encode exported or membrane-tethered proteins are known to play roles in physiological functions (like, acting as substrate-binding proteins and acting as novel ABC transporters) as well as exhibiting virulence factors [36,38]. In almost all the studied strains in this work, the gene encoding Lcp is located downstream of transcriptional regulator of AcrR family. Such regulators are located proximal to alcp gene in all the genomes harbouring lcp or DUF2236-containing domain and they might play role in regulating the expression of lcp genes while bacterial growth on polyisoprene or rubber (Figure 3 - see PDF). The physical map of *N. nova* SH22a lcp and its adjacent genes displays an ORF encoding a α/β-hydrolase like protein upstream to the lcp and an ORF encoding lipoprotein of the ApbE family. This is quite similar to that as observed in the physical map of lcp harboured in Gordoniapolyisoprenivorans VH2 plasmid, p174 [38]. It should be noted that their role in microbial rubber degradation is not clear.

## Presence of virulence factors in the genome sequences:

Genes homologous to those of *M. tuberculosis* with functions related to stress adaptation (sodA, katG and ahpC), phagosome arresting (ndk and ptpA), nitrate reductases (narG, narH, narI and narJ), secreted protein (eis), components of type VII secretion system (eccA, eccB, eccD, mycP andeccCa)and mammalian cell entry (mce) were detected in all the strains (Figure 4; Table S1 - see PDF). Almost all of the studied *Nocardia* strainshave genes related with enhanced intracellular survival protein (eis) whose encoded protein is associated to the intracellular survival of *M. tuberculosis* in macrophage cell lines [65]. The studied genomes from the different *Nocardia* harboured nucleoside diphosphate kinase gene (ndk) which is responsible for the protection of bacterial cells against reactive oxygen species. Basically, nucleoside diphosphate kinase gene (ndk) and protein tyrosine phosphatase (ptpA) have the ability to arrest macrophage phagosomal maturation for the sake of survival and persistence [66,67]. Also, all the considered species of *Nocardia* are found to harbour catalase peroxidase (KatG), superoxide dismutase (SodA) and alkyl hydroperoxide reductase protein C (ahpC) which are known to play role in stress adaptation. The alkyl hydroperoxide reductase protein C helps to resist the peroxynitrite produced by macrophages as a host defensemechanism [68]. Interestingly, as it is known that rubber or polyisoprene materials exhibit the tendency to automatically get oxidized in the presence of atmospheric oxygen and ozone, giving rise to the formation of reactive oxygen species, the presence of SodA and KatG can be supposed to help the *Nocardia* strains in such conditions. A similar condition has been proposed for Gordoniapolyisoprenivorans VH2 [38]. The studied species of *Nocardia* were found to harbour genes encoding respiratory nitrate reductase alpha chain (narG), nitrate reductase beta chain (narH), nitrate reductase gamma chain (narI) and nitrate reductase delta chain (narJ). These are actually believed to be involved in the nitrate reduction pathway and its encoded products are involved in the survival of the opportunistic facultative pathogens during low-oxygen-levels-mediated dormancy in host infections [69,70]. The mammalian cell entry (mce) genes are responsible for coding the MCE-family proteins which are known to have the ability to invade into mammalian cells and survive inside the macrophages [71]. Similarly, the presence of components of type seven secretion system (T7SS)(ccA, eccB,mycP,etc.) in the selected strains of *Nocardia*, exhibit virulence in *M. tuberculosis* indicates that, indicates that they might also be capable of exhibiting similar characteristics [72]. Interestingly, amino acid sequences of virulence factors with functions related to stress adaptation (sodA, katG and ahpC), phagosome arresting (ndk and ptpA), nitrate reductases (narG, narH, narI and narJ), secreted protein (eis), and components of type VII secretion system (eccA, eccB, eccD, mycP and eccCa) from both the rubber degrading strains of *Nocardia* i.e., BSTN01 and SH22a exhibited similar range of percentage identity (Table 2 - see PDF) of the virulence factors exhibited by the other studied pathogenic species of *Nocardia*with respect to that of *M. tuberculosis* (Table S2 - see PDF). There is a report of application of genome editing technology employing CRISPR/Cas9 technique to desirably knockout secondary alcohol dehydrogenase in N. cholesterolicum [73], if such technology is employed in case of rubber degrading microbes, we can expect positive outputs. To cope up with the threat of *Nocardia* contaminated wastes, lytic bacteriophages can be used, as a similar case has been reported for biocontrol of *Nocardia*-stabilized foams in activated sludge plants by lytic bacteriophage GTE2 [74].

## Conclusion:

From the WGS and complete genome sequences of the studied *Nocardia* genomes, it is evident that all the genomes from different *Nocardia* sp. harbour DUF2236 containing domain. This is very apparent that DUF2236 containing domain is conserved in the diverse studied *Nocardia* genomes and is also responsible for encoding the latex clearing protein which is responsible for the primary steps in the process of biodegradation of rubber. Nevertheless, all the studied species of *Nocardia* are also found to harbour virulence factors which are homologous to those of *M. tuberculosis* with functions related to stress adaptation, phagosome arresting, nitrate reductases, secreted protein, effector delivery system and mammalian cell entry (mce). Interestingly, as already discussed above, the mce clusters which were originally identified because of their critical function of invading and surviving inside mammalian cells are now also known to play roles as substrate-binding proteins and mediate the movement of intermediate products formed due to the oxidative cleavage of polyisoprene across the bacterial cell wall. Moreover, the presence of SodA and KatG in rubber degrading *Nocardia* is supposed to help in survival from the oxidative stress due to the formation of activated oxygen species as a result of auto-oxidizing effect of rubber. Such relevant virulent genes in rubber degrading *Nocardia* and the presence of conserved lcp gene or DUF2236 containing domain in facultative human pathogens of *Nocardia* raises a concern in terms of clinical science. A number of clinical products like renal tubes, catheters and central vascular tubes are made up of rubber or latex. Thus, in patients who are immune compromised and are required to undergo treatment employing such rubber/latex products *Nocardia* infection might pose a threat. Such conditions make the studied rubber degrading a potential emerging human pathogen. Further studies would definitely help to better understand any relatedness between the rubber degradation genes and virulence factors if present.

## Funding:

Not applicable

## Authors' contributions:

BS designed and performed the experiment and wrote the main manuscript text; AGM helped acquisition and analysing the in-silico data; SM conceptualized the research, designed and critically revised the manuscript.
